# Development and validation of a novel MR imaging predictor of response to induction chemotherapy in locoregionally advanced nasopharyngeal cancer: a randomized controlled trial substudy (NCT01245959)

**DOI:** 10.1186/s12916-019-1422-6

**Published:** 2019-10-23

**Authors:** Di Dong, Fan Zhang, Lian-Zhen Zhong, Meng-Jie Fang, Cheng-Long Huang, Ji-Jin Yao, Ying Sun, Jie Tian, Jun Ma, Ling-Long Tang

**Affiliations:** 1 CAS Key Laboratory of Molecular Imaging, Institute of Automation, Chinese Academy of Sciences, No. 95 Zhongguancun East Road, Hai Dian District, Beijing, 100190 People’s Republic of China; 2 Department of Radiation oncology, State Key Laboratory of Oncology in South China; Collaborative Innovation Center for Cancer Medicine, Sun Yat-sen University Cancer Center, 651 Dongfeng Road East, Guangzhou, 510060 People’s Republic of China; 3 School of Artificial Intelligence, University of Chinese Academy of Sciences, Beijing, 100049 People’s Republic of China; 4 Department of Radiotherapy, The Fifth Affiliated Hospital, Sun Yat-sen University, Zhuhai, 519000 People’s Republic of China; 5 Beijing Advanced Innovation Center for Big Data-Based Precision Medicine, School of Medicine, Beihang University, Beijing, 100191 People’s Republic of China

**Keywords:** Individualized imaging biomarker, Induction chemotherapy, Survival benefit, Treatment decision, Locoregionally advanced nasopharyngeal cancer

## Abstract

**Background:**

In locoregionally advanced nasopharyngeal carcinoma (LANPC) patients, variance of tumor response to induction chemotherapy (ICT) was observed. We developed and validated a novel imaging biomarker to predict which patients will benefit most from additional ICT compared with chemoradiotherapy (CCRT) alone.

**Methods:**

All patients, including retrospective training (*n* = 254) and prospective randomized controlled validation cohorts (a substudy of NCT01245959, *n* = 248), received ICT+CCRT or CCRT alone. Primary endpoint was failure-free survival (FFS). From the multi-parameter magnetic resonance images of the primary tumor at baseline, 819 quantitative 2D imaging features were extracted. Selected key features (according to their interaction effect between the two treatments) were combined into an Induction Chemotherapy Outcome Score (ICTOS) with a multivariable Cox proportional hazards model using modified covariate method. Kaplan-Meier curves and significance test for treatment interaction were used to evaluate ICTOS, in both cohorts.

**Results:**

Three imaging features were selected and combined into ICTOS to predict treatment outcome for additional ICT. In the matched training cohort, patients with a high ICTOS had higher 3-year and 5-year FFS in ICT+CCRT than CCRT subgroup (69.3% vs. 45.6% for 3-year FFS, and 64.0% vs. 36.5% for 5-year FFS; HR = 0.43, 95% CI = 0.25–0.74, *p* = 0.002), whereas patients with a low ICTOS had no significant difference in FFS between the subgroups (*p* = 0.063), with a significant treatment interaction (*p*_interaction_ <  0.001). This trend was also found in the validation cohort with high (*n* = 73, ICT+CCRT 89.7% and 89.7% vs. CCRT 61.8% and 52.8% at 3-year and 5-year; HR = 0.17, 95% CI = 0.06–0.51, *p* <  0.001) and low ICTOS (*n* = 175, *p* = 0.31), with a significant treatment interaction (*p*_interaction_ = 0.019). Compared with 12.5% and 16.6% absolute benefit in the validation cohort (3-year FFS from 69.9 to 82.4% and 5-year FFS from 63.4 to 80.0% from additional ICT), high ICTOS group in this cohort had 27.9% and 36.9% absolute benefit. Furthermore, no significant survival improvement was found from additional ICT in both groups after stratifying low ICTOS patients into low-risk and high-risks groups, by clinical risk factors.

**Conclusion:**

An imaging biomarker, ICTOS, as proposed, identified patients who were more likely to gain additional survival benefit from ICT+CCRT (high ICTOS), which could influence clinical decisions, such as the indication for ICT treatment.

**Trial registration:**

ClinicalTrials.gov, NCT01245959. Registered 23 November 2010.

## Introduction

Nasopharyngeal carcinoma (NPC) has a unique geographical distribution; 86,700 new cases of nasopharyngeal carcinoma were reported worldwide in 2012 with the highest incidence reported in Southeast Asia [1]. Nearly 70% of newly diagnosed cases of NPC are classified as locoregionally advanced disease [2]. Induction chemotherapy (ICT) followed by concurrent chemoradiotherapy (CCRT) or CCRT alone are both now recommended for locoregionally advanced NPC (LANPC) in the National Comprehensive Cancer Network (NCCN) guideline [3]. Our previous prospective multi-center randomized controlled trial showed that the addition of ICT to CCRT yielded 8–9% improvement in failure-free survival (FFS) in LANPC [4–6]. However, compared to CCRT, extra grade 3 or 4 adverse events, such as neutropenia and leucopenia, were > 40% during ICT [5]. Further analysis of this trial showed that variance of tumor response to ICT was observed in different patients; while 9.1% of patients presented with poor tumor response (stable disease, SD), 11.3% showed complete response (CR) [7]. Limited benefit and obvious toxicity from ICT and differences in response to ICT indicated the need for an individualized biomarker to predict which patients will benefit most from additional ICT.

Tumors are recognized as heterogeneous entities, and the realization that distinct molecular subsets exist requires a shift in cancer treatment development, from a “one-size fits all” to a more personalized and group-based treatment design [8]. Great efforts have been made to search for molecular biomarkers for LANPC, such as plasma Epstein–Barr virus (pEBV)-DNA [9] and miRNAs [10]. However, few biomarkers are widely used as a predictive tool for personalized therapy in clinical practice, and there is therefore an urgent need to identify new biomarkers. As a promising research method for tumor heterogeneity, the role of medical imaging is swiftly evolving from being primarily a diagnostic tool to also include a central role in assisting with individual treatment decision [11].

Radiomics is an emerging technique that can deeply analyze tumor phenotype by converting medical images into minable data and extracting thousands of quantitative features as imaging biomarkers [11]. Noninvasive radiomic features could reflect the pathologic, genetic, and prognostic information of the entire tumor and thus assist with diagnosis and prognosis [12, 13]. Evidence from previous studies showed that radiomic features could predict treatment outcome and screen out patients for individual treatment [14]. For this reason, radiomics provides a possible way to develop clinical decision support systems (CDSSs), individual information-based systems designed to generate patient-specific recommendations, for example, whether a patient is suitable for ICT+CCRT or CCRT alone.

In this study, we aimed to use pretreatment magnetic resonance (MR) images and clinical characteristics of NPC from a retrospective cohort to develop an individualized radiomic biomarker, to predict which patients will benefit most from additional ICT. Then, we used a subset from a randomized controlled trial to validate the performance of the biomarker and to recommend a CDSS the best choice between ICT+CCRT and CCRT alone in LANPC.

## Methods

### Study design and participants

In this study, we included a retrospective cohort for training, and a prospective randomized controlled cohort for validation, all from Sun Yat-sen University Cancer Center (Guangzhou, China). The validation cohort was a subset of our previous multi-center, open-label, stratified randomized phase 3 controlled trial (ClinicalTrials.gov identifier NCT01245959) comparing ICT+CCRT and CCRT alone in patients between March 2011 and August 2013 [5], which was an intention-to-treat population. For the training cohort, we retrospectively retrieved medical records of LANPC patients who received radical ICT+CCRT or CCRT between 2008 and 2016. Patients who met the inclusion and exclusion criteria and treatment, in accordance with our previous open-label, phase 3 randomized controlled trial, were recruited in the training cohort (detailed in Additional file 1). The authenticity of this study has been validated by uploading the key raw data onto the Research Data Deposit public platform (www.researchdata.org.cn), with the approval RDD number as RDDA2019001070. Finally, 254 and 248 patients were included in the training and validation cohorts, respectively. 

The ethical review board of our institution approved this retrospective analysis of anonymous data, and the requirement for informed consent was waived. All patients underwent pretreatment multi-parametric magnetic resonance imaging (MRI) within 2 weeks before any anti-cancer treatment. Non-contrast enhanced MRI (T1-weighted images in axial, coronal, and sagittal planes, as well as axial T2-weighted images) and contrast-enhanced sequence (axial and sagittal T1-weighted images, as well as coronal T1-weighted images) were performed sequentially for each patient. Detailed MR protocols (version of MR scanner, magnet strengths, contrast agents, image thickness, etc.) are shown in Additional file 2, Additional file 10: Table S1, and Additional file 11: Table S2.

The radiotherapy and chemotherapy treatment details have been reported previously [5]. After treatment, patients were assessed every 3 months during the first 3 years, and every 6 months thereafter. The primary endpoint was FFS (time to locoregional failure, distant failure, or death from any cause, whichever occurred first). The secondary endpoints included overall survival ([OS], time to death from any cause), distant FFS ([D-FFS], time to distant failure), and locoregional FFS ([LR-FFS], time to local or regional failure or both).

Before the model construction, there should be no subjective judgment by doctors, and patients should have equal chance to choose ICT+CCRT or CCRT alone, when using observational data to evaluate the treatment effects. Therefore, we used Inverse probability of treatment weighting (IPTW) to balance the baseline differences in the training cohort [15]. IPTW was performed on the basis of age, sex, pEBV DNA, cervical nodal necrosis, primary tumor volume, N stage, and T stage (detailed description is shown in Additional file 3). Matched patients were included in the analysis, as the matched training cohort.

### Procedures

**Fig. 1:**
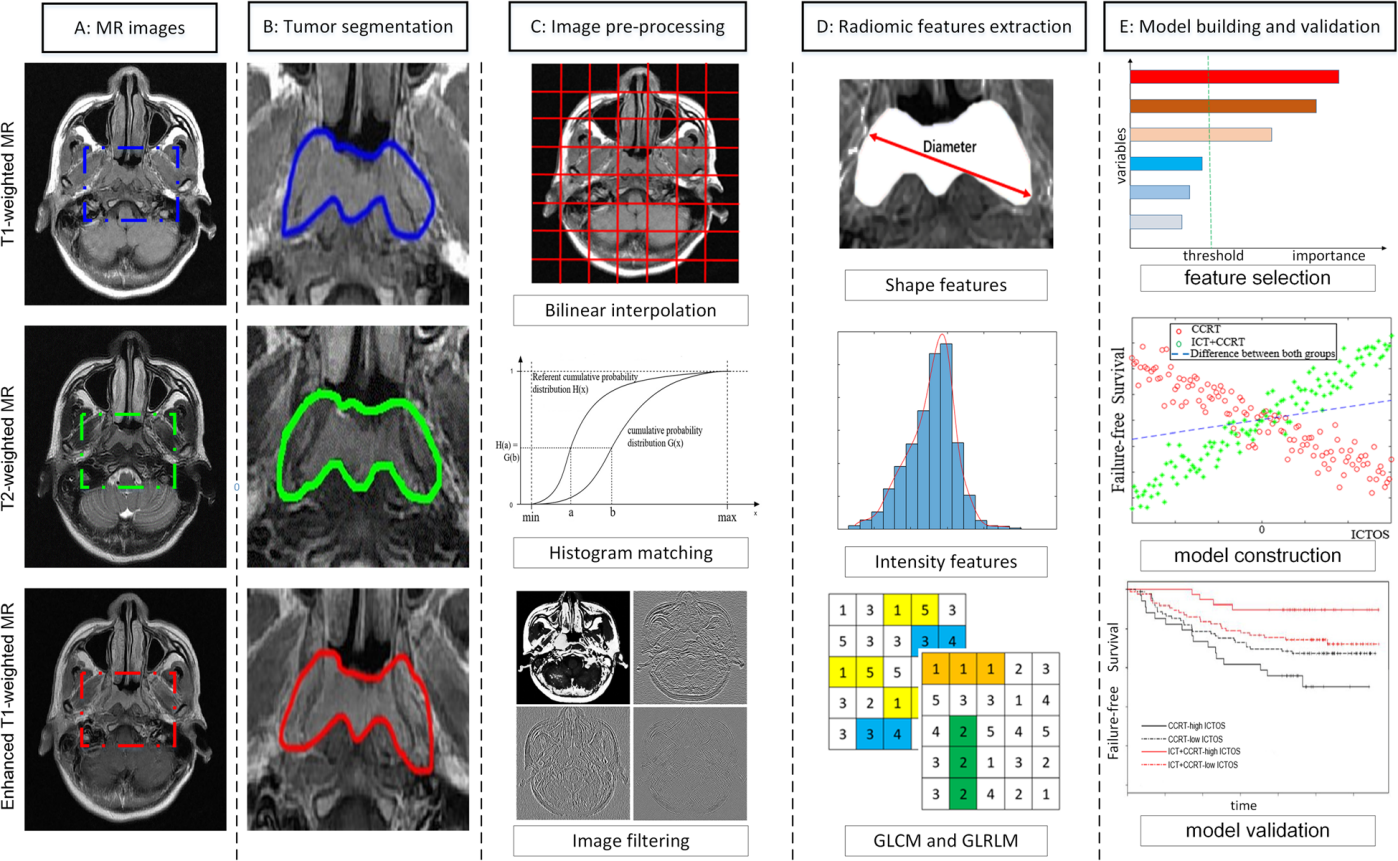
Radiomics workflow in this study. **a** Collection of multi-sequence MR images. **b** Tumor segmentation by radiologists. **c** Preprocessing of the MR images. **d** Feature extraction from tumor region. **e** Model construction and validation

The radiomics workflow is shown in Fig. 1. All MR images were retrieved from the picture archiving and communication system (PACS) and exported to the ITK-SNAP software (version 3.4.0; www.itksnap.org) for manual segmentation. The region of interest (ROI) was delineated manually in the primary tumor on each slice of the axial T1-weighted, T2-weighted, and contrast-enhanced T1-weighted images. Coronal and sagittal images were only used to guide the cross-sectional segmentation of the ROIs. Therefore, there were three different ROIs segmented for each patient in this study. A radiologist with 10 years of experience with MR (L.L.T.) performed all image segmentations. To evaluate the reproducibility of feature among segmentations by different radiologists (inter-observer) or one radiologist at different times (intra-observer), 30 patients were randomly selected 30 days after the initial segmentation and their images were segmented again by L.L.T. and another radiologist (F.Z.) in the same way. All the radiologists were blinded to treatment group and patient outcome.

After segmentation, interpolation and normalization of MRI images were performed to control inter-scanner and inter-vendor variability of features (detailed in Additional file 3). Then, 273 2D radiomic features were extracted from each ROI, and a total of 819 2D features were achieved per patient. All the features have been referred to in the image biomarker standardization initiative (IBSI) formula [16] and in the study by Aerts et al. [17]. These features were derived from the tumor image intensity, shape, gray-level co-occurrence matrix (GLCM), and gray-level run-length matrix (GLRLM) (Additional file 3) [16, 17]. The process of feature extraction was conducted using Matlab 2017b (Mathworks, Natick, MA, USA). Radiomic features of all patients were standardized by the *z*-score method, based on parameters calculated from the training cohort. Then, the intra-observer/inter-observer agreements of radiomic features were assessed using the inter-class correlation coefficient (ICC) on the 30 patients’ multiple segmentation data [18]. For the intra-observer agreement, the ICC of each radiomic feature was calculated between the twice segmentations by L.L.T. For the inter-observer agreement, the ICC of each radiomic feature was calculated between segmentations by L.L.T. and F.Z. The features with ICCs > 0.8 in both intra-observer and inter-observer tests were reserved.

To construct the predictive model, we first identified which factors could provide different prognostic information in the ICT+CCRT and CCRT alone groups. Using the matched training cohort, the interaction term of treatment and each radiomic feature was ranked by its univariate *p*_interaction_ value in a Cox proportional hazard model. Radiomic features were excluded when their correlation coefficient with the former ranked features was > 0.8, to reduce redundancy between radiomic features. The remaining radiomic features were used to train a multivariable Cox proportional hazard model, with FFS as the endpoint, using the modified covariate method [19]. Backward step-wise selection was used to select variables whose coefficients reached statistical significance. The outcome score of the model, termed Induction Chemotherapy Outcome Score (ICTOS), was calculated using the difference in treatment outcome between ICT+CCRT and CCRT alone. Patients with scores > 0 (high ICTOS) benefit from additional ICT, while patients with scores ≤ 0 (low ICTOS) do not benefit from additional ICT. The “0” cutoff point represents the no difference point in predicted outcomes for additional ICT. Stratified analyses for low ICTOS patients were performed to test the ICTOS’s robustness in various subgroups of the validation dataset and find those patients who need further treatment.

### Statistics

We aimed to identify patients who will benefit the most, of the entire cohort, defined as the population who would have a 5-year FFS rate increase from 65% (with CCRT alone) to 90% (with ICT+CCRT) (target HR < 0.25) [4]. The statistical methodology is as reported by Zhao et al. [20]. With 110 patients and 29 events, 1:1 assigned to each arm and 5% two-sided statistical significance, the power for the superior test was 0.96. Assuming this population accounts for 47% of LANPC [21], 234 patients were required for the training cohort.

Kaplan-Meier curves were generated for FFS (the primary endpoint). All hazard ratios (HRs), 3-year FFS, and 5-year FFS were reported with 95% confidence intervals (CIs). We used the Greenwood’s formula to obtain the 95% CI for 3-year and 5-year FFS. Wald test was used to assess the significance of the interaction term while log-rank test was used to compare the different survival curves.

Continuous variables were expressed as median (range), and group comparison was performed by either the *t* test or the Wilcoxon rank-sum test. Categorical variables were expressed as percentages, and group comparison was performed by either the Pearson *χ*^2^ test or the Fisher exact test. Statistical analysis was conducted with R software (version 3.4.3; http://www.Rproject.org). Detailed description of the modified covariate method is shown in Additional file 3. A two-sided *p* value < 0.05 was used to indicate statistical significance. Note that only absolute benefits in this manuscript were expressed as percentage points, and all others were expressed as percentages.

## Results

**Fig. 2:**
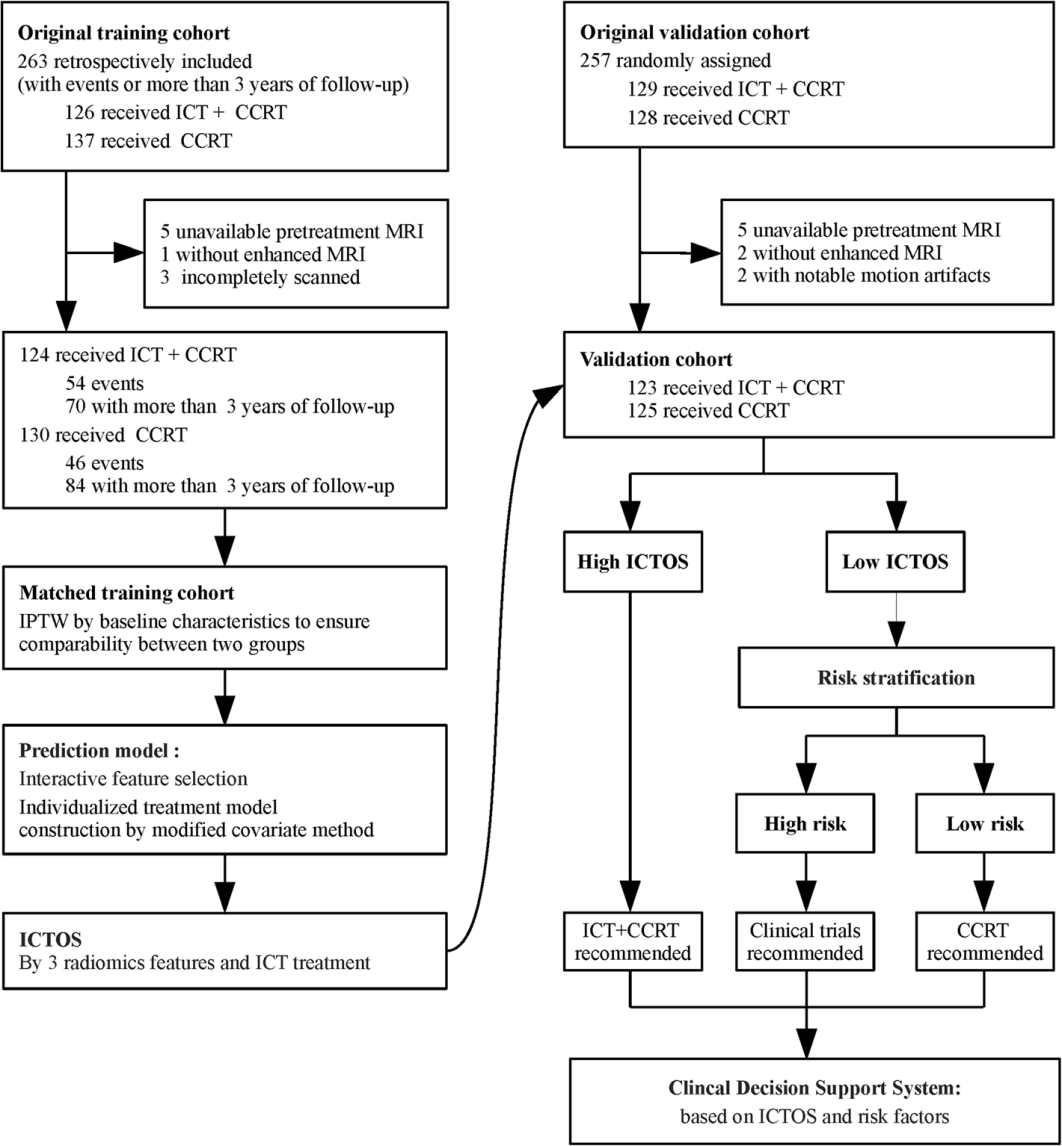
Study design. The belief design of patient recruitment, matching procedures, model construction and validation, and further stratification analysis. ICT, induction chemotherapy; CCRT, concurrent chemoradiotherapy; MRI, magnetic resonance imaging; IPTW, inverse probability of treatment weighting; ICTOS, Induction Chemotherapy Outcomes Score

Finally, 254 and 248 patients (from the initial 263 and 257 eligible patients) were included in the training and validation cohorts, respectively (Fig. 2). After IPTW, the ICT+CCRT and CCRT alone groups achieved good balance in the matched training cohort (matched sample size *n* = 247.8). The characteristics of the matched training cohort are shown in Additional file 12: Table S3 and Additional file 5: Figure S1. In the matched training and validation cohorts, respectively, 39.4% and 28.6% patients had treatment failure or died.

After the intra-observer/inter-observer agreement assessment, 689 radiomic features with ICC ≥ 0.8 were reserved for subsequent analysis (Additional file 6: Figure S2). In the matched training cohort, three radiomic features including intensity (skewness) and two texture (variance from Gray-Level Co-occurrence Matrix [GLCM_variance] and Long Run High Gray Level Emphasis from Gray-Level Run-Length matrix [GLRLM_LRHGLE]) features were selected and incorporated into the ICTOS model: − 0.668*skewness - 0.442*GLCM_variance + 0.410*GLRLM_LRHGLE. All three features are from contrast-enhanced T1-weighted sequence with the formulae used in their calculation shown in Additional file 4.

**Fig. 3:**
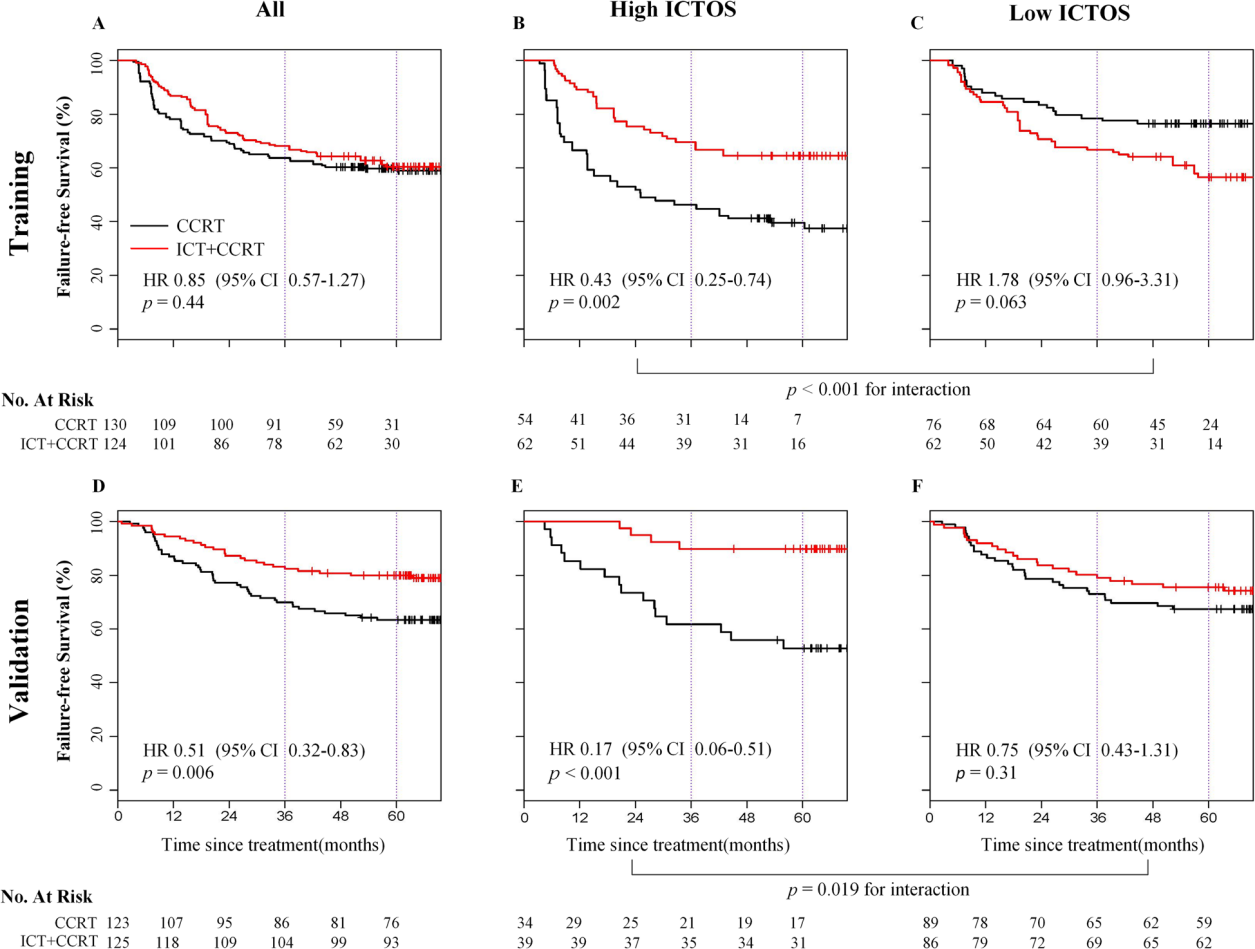
Failure-free survival in the training and validation cohorts stratified by low and high ICTOS. Kaplan-Meier curves compared the ICT+CCRT patients and CCRT patients in the whole group (**a**), high ICTOS group (**b**), and low ICTOS group (**c**) in the matched training cohort. Kaplan-Meier curves compared the ICT+CCRT patients and CCRT patients in the whole group (**d**), high ICTOS group (**e**), and low ICTOS group (**f**) in the prospective validation cohort. ICT, induction chemotherapy; CCRT, concurrent chemoradiotherapy; ICTOS, Induction Chemotherapy Outcomes Score; HR, hazard ratio; CI, confidence interval

In the matched training cohort, 3-year and 5-year FFS were 68.2% (95% CI = 0.60–0.77) vs. 63.7% (95% CI = 0.56–0.73) and 59.4% (95% CI = 0.51–0.70) vs. 59.0% (95% CI = 0.51–0.69) for ICT+CCRT and CCRT alone patients, respectively (HR = 0.85, 95% CI = 0.57–1.27, *p* = 0.44; Fig. 3a). Among patients, 116.2 (46.9%) had high ICTOS while 131.6 (53.1%) had low ICTOS. Note that the patient numbers here are not integers because they are weighted numbers of the matched training cohort after IPTW. ICTOS could predict treatment outcome for the additional ICT (*p*_interaction_ < 0.001). In the high ICTOS group, 3-year and 5-year FFS were 69.3% (95% CI = 0.59–0.82) and 64.0% (95% CI = 0.53–0.78) in ICT+CCRT subgroup and 45.6% (95% CI = 0.34–0.61) and 36.5% (95% CI = 0.25–0.53) in CCRT alone subgroup (HR = 0.43, 95% CI = 0.25–0.74, *p* = 0.002, Fig. 3b). Therefore, in the high ICTOS group, there were 23.7% absolute benefit at 3-year in favor of ICT+CCRT vs. CCRT alone, and 27.5% absolute benefit at 5-year. While in the low ICTOS group, 3-year and 5-year FFS were 67.1% (95% CI = 0.57–0.80) and 55.8% (95% CI = 0.44–0.71) in the ICT+CCRT subgroup and 78.7% (95% CI = 0.70–0.89) and 76.5% (95% CI = 0.67–0.87) in the CCRT alone subgroup (HR = 1.78, 95% CI = 0.96–3.31, *p* = 0.063; Fig. 3c).

In the validation cohort, the last follow-up was on August 31, 2018 [6]. The median follow-up was 69.5 months (IQR 61.9–76.9). The 3-year and 5-year FFS were 82.4% (95% CI = 0.76–0.89) vs. 69.9 (95% CI = 0.62–0.79), and 80.0% (95% CI = 0.73–0.87) vs. 63.4% (95% CI = 0.55–0.73) for ICT+CCRT and CCRT alone patients, respectively (HR = 0.51, 95% CI = 0.32–0.83, *p* = 0.006, 12.5% and 16.6% absolute benefit at 3 and 5 years in favor of ICT+CCRT vs. CCRT alone; Fig. 3d). ICTOS identification ability was validated in our validation cohort (*p*_interaction_ = 0.019). In the high ICTOS group (*n* = 73/248), 3-year and 5-year FFS were 89.7% (95% CI = 0.81–1.00) and 89.7% (95% CI = 0.81–1.00) in ICT+CCRT; and 61.8% (95% CI = 0.47–0.81) and 52.8% (95% CI = 0.38–0.73) in CCRT alone subgroups (HR = 0.17, 95% CI = 0.06–0.51, *p* < 0.001, Fig. 3e). Therefore, in the high ICTOS group, there were 27.9% absolute benefit at 3-year in favor of ICT+CCRT vs. CCRT alone, and 36.9% absolute benefit at 5-year. While in the low ICTOS group (*n* = 175/248), 3-year and 5-year FFS were 79.1% (95% CI = 0.71–0.88) and 75.5% (95% CI = 0.67–0.85) in the ICT+CCRT, and 73.0% (95% CI = 0.64–0.83) and 67.4% (95% CI = 0.58–0.78) in the CCRT alone subgroups (HR = 0.75, 95% CI = 0.43–1.31, *p* = 0.31; Fig. 3f).

Clinical risk factors did not show consistently significant associations with ICTOS across training and validation cohorts (Table 1). Furthermore, with secondary endpoints (OS, D-FFS, and LR-FFS), ICTOS also successfully selected patients who could benefit most from additional ICT (Additional file 7: Figure S3), demonstrating the good generalizability of our model. The interaction between additional ICT and prognostic factors including pEBV DNA (< 2000 vs. ≥ 2000 copy/ml), N stage (N1 vs. N2–3), and T stage (T1–3 vs. T4) was not significant in the matched training (*p*_interaction_ = 0.73 for pEBV DNA, *p*_interaction_ = 0.21 for N stage, *p*_interaction_ = 0.071 for T stage) and validation (*p*_interaction_ = 0.74 for pEBV DNA, *p*_interaction_ = 0.47 for N stage, *p*_interaction_ = 0.77 for T stage) cohorts (Additional file 8: Figure S4). Moreover, in an interaction analysis for pEBV DNA with 1500, 4000, and 6000 copy/ml as cutoff points, all *p*_interaction_ values remained non-significant. These results illustrate that these factors were not predictive of treatment outcome for the additional ICT despite some being prognostic factors [22].

**Table 1:** Association of baseline characteristics with ICTOS in both training and validation sets

	Training set					
	Low ICTOS (*n* = 138)	High ICTOS (*n* = 116)	*P* value	Low ICTOS (*n* = 175)	High ICTOS (*n* = 73)	*P* value
Age			0.06	0.49		0.49
< 42 years	45 (32.6)	52 (44.8)		81 (46.3)	38 (52.1)	
≥ 42 years	93 (67.4)	64 (55.2)		94 (53.7)	35 (47.9)	
Sex			0.02			0.40
Male	109 (79.0)	75 (64.7)		144 (82.3)	56 (76.7)	
Female	29 (21.0)	41 (35.3)		31 (17.7)	17 (23.3)	
Staging^a^			0.04			0.80
III	84 (70.9)	55 (47.4)		95 (65.7)	46 (73.0)	
IV	54 (39.1)	61 (52.6)		60 (34.3)	27 (37.0)	
T stage^a^			0.21			0.33
T1	4 (2.9)	2 (1.7)		5 (2.9)	1 (1.4)	
T2	14 (10.1)	7 (6.0)		8 (4.6)	5 (6.8)	
T3	82 (59.4)	62 (53.4)		114 (65.1)	40 (54.8)	
T4	38 (27.5)	45 (38.8)		48 (27.4)	27 (37.0)	
N stage^a^			0.48	0.08		0.08
N1	66 (47.8)	47 (40.5)		98 (56.0)	36 (49.3)	
N2	50 (36.2)	46 (39.7)		62 (35.4)	35 (47.9)	
N3	22 (15.9)	23 (19.8)		15 (8.6)	2 (2.7)	
Cervical nodal necrosis			0.90			0.32
No	99 (71.7)	85 (73.3)		114 (65.1)	53 (72.6)	
Yes	39 (28.3)	31 (26.7)		61 (34.9)	20 (27.4)	
Primary tumor volume	< 0.001		< 0.001	0.56		0.56
< 34 ml	97 (70.3)	53 (45.7)		88 (50.3)	33 (45.2)	
≥ 34 ml	41 (29.7)	63 (54.3)		87 (49.7)	40 (54.8)	
Pretreatment pEBV DNA level			0.17			0.08
< 2000 copy/ml	75 (54.3)	52 (44.8)		60 (45.7)	24 (32.9)	
≥ 2000 copy/ml	63 (45.7)	64 (55.2)		95 (54.3)	49 (67.1)	
Chemoradiotherapy regimen			0.22			0.64
ICT+CCRT	62 (44.9)	62 (53.4)		86 (49.1)	39 (53.4)	
CCRT	76 (55.1)	54 (46.6)		89 (50.9)	34 (46.6)	

*ICTOS* Induction Chemotherapy Outcomes Score, *pEBV DNA* plasma Epstein–Barr Virus DNA, *CCRT* concurrent chemoradiotherapy, ICT, induction chemotherapy

^a^Staging, T classification, and N classification were based on the 7th edition of the American Joint Committee on Cancer/International Union Against Cancer staging systems

**Fig. 4:**
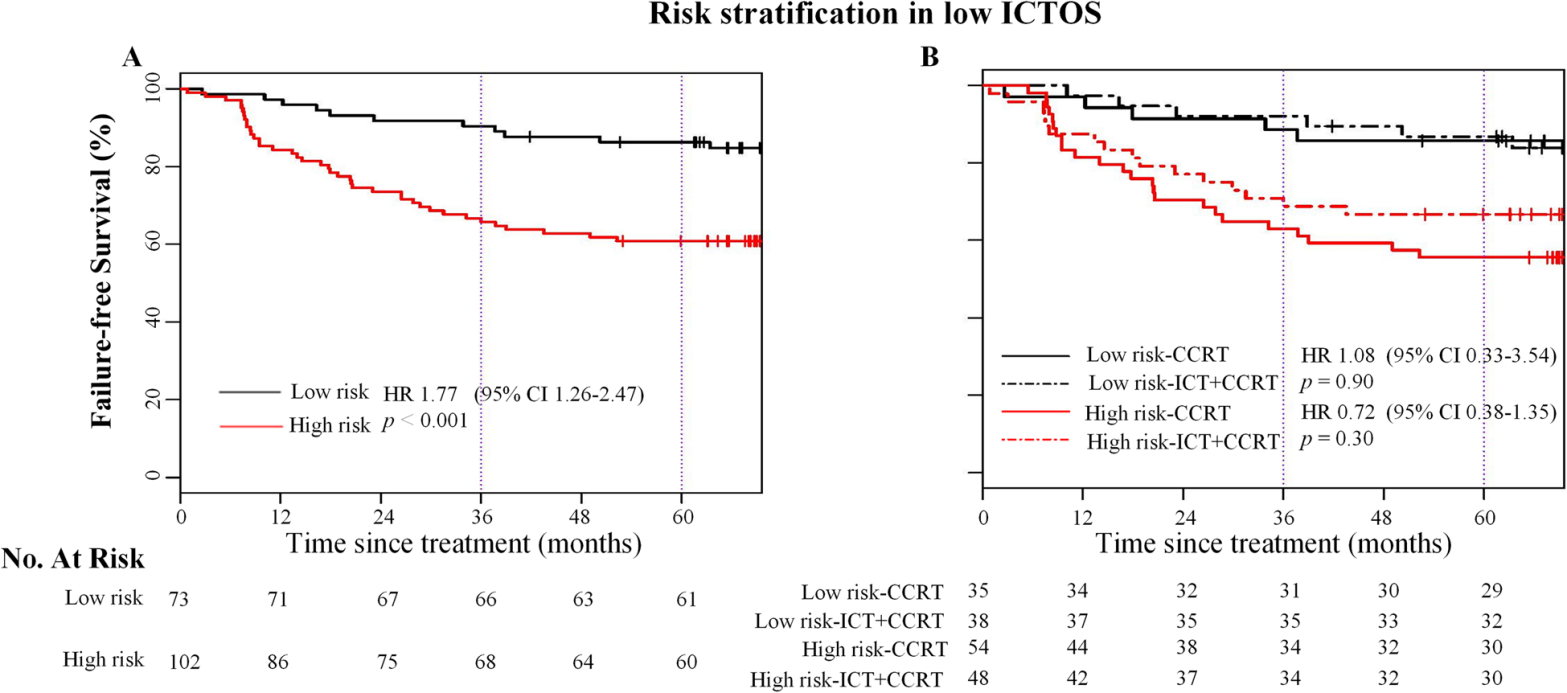
Stratification analysis of patients with low ICTOS in the prospective validation cohort. Kaplan-Meier curves compared the low-risk and high-risk patients (**a**). Kaplan-Meier curves compared the ICT+CCRT patients and CCRT patients in the low-risk and high-risk groups (**b**). ICT, induction chemotherapy; CCRT, concurrent chemoradiotherapy; ICTOS, Induction Chemotherapy Outcomes Score; HR, hazard ratio; CI, confidence interval

Since low ICTOS patients in the validation cohort could not gain significant FFS improvement from the additional ICT, it was not possible to conclude that CCRT alone was sufficient in patients with unsatisfactory 5-year FFS (71.4%, 95% CI = 0.65–0.78). Therefore, we further explored the low ICTOS group by conducting risk stratification. N stage, T stage, primary tumor volume, and pEBV DNA were confirmed as stratification factors (see detailed results in Additional file 13: Table S4). To facilitate clinical application, patients with more than two high-risk factors were stratified into the high-risk group, and others into the low-risk group. Low-risk group patients had significantly higher 3-year and 5-year FFS than high-risk group (90.4% [95% CI = 0.84–0.97] vs. 65.7% [95% CI = 0.57–0.76], 86.3% [95% CI = 0.79–0.95] vs. 60.8% [95% CI = 0.52–0.71]; HR = 1.77, 95% CI = 1.26–2.47, *p* < 0.001, Fig. 4a). In both groups, there was no significant difference in survival curves between ICT+CCRT and CCRT alone patients (low-risk group, HR = 1.08, 95% CI = 0.33–3.54, *p* = 0.90; high-risk group, HR = 0.72, 95% CI = 0.38–1.35, *p* = 0.30, Fig. 4b), which confirmed the robustness of our model in different subgroups.

## Discussion

In this relatively large-cohort study, we developed an individualized MRI biomarker, ICTOS, which could predict treatment outcome for additional ICT. The constructed biomarker showed impressive performance in the validation cohort from a prospective randomized controlled trial (NCT01245959). Moreover, in comparison to the widely used clinical risk factors, our proposed biomarker was more effective. Furthermore, combining the clinical prognostic factors with ICTOS, we built CDSS, which might predict the subgroups that would benefit most from ICT+CCRT or CCRT alone.

Interestingly, our model successfully identified patients who would benefit most from ICT+CCRT in the prospective validation cohort; high ICTOS patients from ICT+CCRT subgroup had nearly 90% 5-year FFS with 36.9% absolute benefit, whereas, without ICTOS identification, ICT+CCRT group had 80.0% 5-year FFS with a 16.6% absolute benefit.

According to NCCN guideline, ICT+CCRT is recommended for NPC patients with stages II-IVa (accounting for > 90% of all non-metastasis NPC) [23]. However, there was no biomarker discovered for selecting patients who could benefit most from ICT+CCRT based on individual tumor characteristics. Indeed, the development of truly predictive (as opposed to prognostic) treatment response biomarker is a difficult proposition, which ideally requires balanced datasets from treated versus untreated patients. Our previous randomized clinical trials were perfectly suited for this purpose. Our results showed that additional ICT could improve FFS in high, but not in low, ICTOS patients. The result indicated that high ICTOS patients benefited the most from additional ICT; thus, ICT+CCRT should be recommended for them. Our MRI radiomic-based ICTOS, the first validated imaging biomarker developed to predict treatment outcome for therapy in NPC, is potentially a useful tool clinically, for specifically selecting patients for ICT.

Three 2D radiomic features (skewness, GLCM_variance, and GLRLM_LRHGLE) were finally selected to construct ICTOS to predict benefits from additional ICT. Interestingly, all three features were extracted from contrast-enhanced T1 MR images profiling the perfusion and permeability of tumor vasculature. This finding suggests that tumor angiogenesis might be the predominant factor for the true therapeutic gain, which is in accordance with preliminary studies on mechanism of tumor resistance to chemotherapy [24]. We selected several typical images, with different levels of the three radiomic features and attempted surmising the biologic mechanism of these image features (Additional file 9: Figure S5). Skewness indicates the bias in contrast enhancement correlating with vasculature density, and thus blood supply, suggesting that patients with low ICTOS might have impaired drug delivery, resulting in ineffective ICT [24]. Conversely, the remaining two features represent tumor vasculature heterogeneity. Low ICTOS suggested a high heterogeneity in the structure and function of tumor vasculature. This might lead to a more hostile and complex tumor microenvironment with hypoxia, acidosis, and promotion of the tumor population towards more chemo-resistant one, therefore compromising ICT efficacy [24]. Our findings are also consistent with features found in a previous study predicting the short-term response to ICT in NPC [25]. A preliminary trial (RTOG 0615) [26] showed a promising response by adding bevacizumab to standard chemoradiation in LANPC. The spatially explicit analysis of radiomic feature to tumor vasculature, the mechanism of tumor angiogenesis to chemo-resistance, and the confirmation of the chemotherapeutic gain from induction of vessel normalization in NPC still warrants further investigation.

As a key classification of the anatomical extent of tumor, the current TNM staging system reflecting tumor size (T stage), lymph node status (N stage), and metastasis status (M stage) is the only tool to guide patient selection for ICT in NPC [27]. In our study, prognostic factors including pEBV DNA, N stage, and T stage were not predictive of treatment outcome for the additional ICT. The above prognostic factors were mainly related to tumor burden, but not intrinsic chemosensitivity. In this study, only LANPC patients were included; obviously, the difference in tumor load among patients, being relatively slight, might be concealed by more powerful treatment response difference from our imaging biomarker; hence, these prognostic factors in the LANPC could not distinguish who benefited most from ICT.

Our subgroup analysis showed that patients with low ICTOS and high risk had the worst prognosis (5-year FFS rate in these patients was only 60.8%). Possible reasons included advanced N and T stages, as well as large tumor volume, which were all proved to be negative prognostic factors. In this subgroup, the introduction of ICT was ineffective and the treatment outcome with CCRT alone was poor. Thus, the patients in this subgroup might need intensification of therapy or new therapies, such as anti-angiogenesis [26], metronomic adjuvant chemotherapy [28], and immunotherapy [29]. While for the patients with low ICTOS and low risk, CCRT alone already had a good 5-year FFS of 86.3% and the introduction of ICT generated no significant benefit. Therefore, CCRT alone might be preferred for patients in this subgroup to avoid unnecessary ICT-related expense and toxicity, as well as the prolonged wait before definitive radiotherapy. In conclusion, our CDSS, which combined radiomic ICTOS with clinical prognostic factors, could classify advanced locoregional NPC into three groups with appropriate recommendations for individual therapies as follows: (1) high ICTOS: ICT+CCRT, (2) low ICTOS and low risk: CCRT alone, and (3) low ICTOS and high risk: clinical trials. This is the first time that clinical prognostic and predictive factors have been integrated to guide the individualized treatment of NPC. However, further validation in larger population is still required for the latter two recommendations.

The generalizability of this imaging biomarker is a major concern in clinical practice. For NPC patients, MRI is a routine image method for staging, because of its superiority in soft-tissue contrast. Via target delineation in radiotherapy, segmentation of tumor is unlikely to bring extra burden to clinical practice. Moreover, the imaging biomarker can be automatically generated simply by importing ICTOS-based module into treatment planning system. Besides, through the stratification analysis, our imaging biomarker was not affected by clinical factors like stage and pEBV DNA, showing the potential for generalization. We have deposited our biomarker and CDSS into open access (www.radiomics.net.cn/platform.html) to facilitate the validation and application.

Our study had some limitations. First, due to the retrospective nature of the training set, inevitably, there was possible selection bias between the two treatment groups in the training cohort. This could also be found when comparing the survival results between the training and validation cohorts (Fig. 3a, d), the higher event rate of ICT group in training cohort than the validation cohort. However, this high event rate in the training cohort allowed us to train the ICTOS with a small number of patients. Moreover, validation in the subset of a randomized controlled dataset indicated the robustness of ICTOS. Second, due to the limitation of the data size, further investigations in additional independent cohorts are necessary. For example, larger and multi-center cohorts, as well as our ongoing trials with another regimen of ICT (NCT01872962, gemcitabine and cisplatin), could provide potential validation cohorts to test the reproducibility and universality of ICTOS. Third, our previous work found that the expression of 13 genes [30] predicted the prognosis and efficacy of CCRT in LANPC. Other studies proposed proteins, such as SQSTM1 [21] and genes, such as ERCC1 [31] to predict chemotherapy response in NPC. In the following study, we plan to incorporate the molecular profile based on the same settings (LANPC patients) to confirm the current findings. In our study, many different MR scanners and MR acquisition protocols were used; how this influenced the radiomic features and ICTOS model should be studied further in the future.

In conclusion, the proposed imaging biomarker (ICTOS) provided a robust, feasible, and economical solution to identify patients who were more likely to gain a survival benefit from ICT+CCRT, which could influence clinical decisions such as the indication for ICT treatment.

## Supplementary information

**Additional file 1.** The inclusion and extrusion criteria.

**Additional file 2.** Magnetic resonance image acquisition parameters.

**Additional file 3.** Supplementary methods.

**Additional file 4.** ICTOS calculation formula.

**Additional file 5: Figure S1.** Performance of inverse probability of treatment weighting between ICT+CCRT and CCRT patient groups. Abbreviations: CCRT, concurrent chemoradiotherapy; ICT, induction chemotherapy; IPTW, Inverse Probability of Treatment Weighting.

**Additional file 6: Figure S2.** Analysis of radiomic features’ robustness. Abbreviations: T1, T1-weighted images; T2, T2-weighted images; T1C, contrast enhanced T1-weighted images; ICC, inter-class correlation coefficient. Note: An ICC greater than 0.8 indicates good consistency. As the figure shown, a large majority of features achieve this standard, which affirm the reproducibility of imaging features.

**Additional file 7: Figure S3.** Kaplan-Meier survival curves with secondary endpoints for the two treatment groups in the validation cohort. Abbreviations: ICT, induction chemotherapy; CCRT, concurrent chemoradiotherapy; ICTOS, Induction Chemotherapy Outcomes Score; HR, hazard ratio; CI, confidence interval. Note: Because there was no patient with distance failure in the high ICTOS group, relative measurements could not be calculated (E).

**Additional file 8: Figure S4.** Failure-free survival in the validation cohort stratified by the usual clinical risk factors. Abbreviations: ICT, induction chemotherapy; CCRT, concurrent chemoradiotherapy; pEBV DNA, plasma Epstein–Barr Virus DNA. Note: *P*_interaction_ = 0.74 for pEBV DNA, *P*_interaction_ = 0.47 for N stage, *P*_interaction_ = 0.77 for T stage. ^a^Staging, T classification, N classification were determined based on the 7th edition of the American Joint Commission on Cancer staging system.

**Additional file 9: Figure S5.** MR images of patients with high ICTOS and low ICTOS. Abbreviations: MR, Magnetic Resonance; ICTOS, Induction Chemotherapy Outcomes Score. Note: Contrast enhanced T1 MRI images in a patient with high ICTOS (skewness = -2.02; GLRLM_LRHGLE = 4.58; GLCM_variance = -1.26, ICTOS = 3.80, Figure A); and a patient with low ICTOS (skewness = 1.82; GLRLM_LRHGLE = -1.46; GLCM_variance = 1.37, ICTOS = -2.49, Fig. B).

**Additional file 10: Table S1.** MR imaging sequences.

**Additional file 11: Table S2.** Acquisitions parameters of axial CE-T1W FSE in the training and validation cohorts.

**Additional file 12: Table S3.** Standardized differences in the training sets after weighting by propensity score.

**Additional file 13: Table S4.** Univariate analyses of risk factors in patients with low ICTOS (*n* = 175).

## Data Availability

The datasets used and/or analyzed during the current study are available from the corresponding author on reasonable request.

## Abbreviations

CCRT: Concurrent chemoradiotherapy
CDSSs: Clinical decision support systems
CI: Confidence interval
CR: Complete response
D-FFS: Distant failure-free survival
FFS: Failure-free survival
GLCM_variance: Variance from Gray-Level Co-occurrence Matrix
GLRLM_LRHGLE: Long Run High Gray Level Emphasis from Gray-Level Run-Length matrix
HR: Hazard ratio
ICT: Induction chemotherapy
ICTOS: Induction Chemotherapy Outcome Score
IPTW: Inverse probability of treatment weighting
IQR: Interquartile range
LANPC: Locoregionally advanced nasopharyngeal carcinoma
LR-FFS: Locoregional failure-free survival
MR: Magnetic resonance imaging
NCCN: National Comprehensive Cancer Network
NPC: Nasopharyngeal carcinoma
OS: Overall survival
PACS: Picture archiving and communication system
pEBV: Plasmas Epstein–Barr virus
ROI: Region of interest
SD: Stable disease
