# Supplementary material for: Development and validation of a novel MR imaging predictor of response to induction chemotherapy in locoregionally advanced nasopharyngeal cancer: a randomized controlled trial substudy (NCT01245959)
Source: BMC Med. 2019 Oct 23;17:190. doi: 10.1186/s12916-019-1422-6 (PMC6806559; doi:10.1186/s12916-019-1422-6)
Supplement: Supplementary file 1 — Additional file 1. The inclusion and extrusion criteria. [file 12916_2019_1422_MOESM1_ESM.docx]

**Appendix S1: The inclusion and extrusion criteria**

This study included a retrospective cohort for training and a subgroup from the prospective randomized controlled trial (NCT01245959) for validation. The inclusion and extrusion criteria of our study are as follows.

**Inclusion criteria for the training set:**

1) Baseline characteristics and treatment were in accordance with our previous open-label, phase 3 randomized controlled trial, which briefly included the following patients:

1. New histologically confirmed and non-distant metastasis;
2. Nonkeratinizing stage III to stage IVB NPC (except T3-4N0) reassessed according to the 7th edition of the American Joint Committee on Cancer/International Union Against Cancer staging systems (*1*);
3. Who were aged 18 to 60 years, with Karnofsky performance scores > 70;
4. Without previous chemotherapy or RT or other malignant tumors;
5. Who received either induction chemotherapy (ICT) followed by concurrent chemoradiotherapy (CCRT) or CCRT alone: three cycles of cisplatin, fluorouracil, and docetaxel (TPF) chemotherapy as docetaxel and cisplatin intravenously on day 1, and fluorouracil on days 1–5 (120 h infusion) 3-weekly before radiotherapy in the ICT+CCRT group; two to three cycles of concurrent chemotherapy as cisplatin intravenously every 3 weeks during radiotherapy, in both groups. Note that, all patients finished the complete treatment of ICT+ CCRT or CCRT alone in the training cohort.
6. Who did not receive adjuvant chemotherapy, targeted therapy, or biotherapy during the course of radical treatment.
7. Who received cumulative radiation doses as ≥ 66 Gy to the primary tumor and ≥ 50 Gy to the bilateral cervical lymph nodes and potential sites of local infiltration, using intensity-modulated radiotherapy (IMRT).

2) Based on the aim of the current study, we further required the following criteria for the training set:

1. separate from the abovementioned trial;
2. with treatment failure (locoregional recurrences and/or distant metastases) or without treatment failure but with > 3-year follow-up;
3. with pretreatment MRI within 2 weeks before treatment and pEBV-DNA level.

**Exclusion criteria for the training set:**

1. Incomplete pretreatment MRI of head and neck including axial CET1-w, T1-w, and T2-w images.
2. Notable motion artifacts in the axial CET1-w, T1-w, and T2-w images.

Note that, the validation cohort was from the intention-to-treat population in our previous randomized controlled trial (ClinicalTrials.gov identifier NCT01245959) [2], which contained some patients who did not complete the treatment. Our former trial NCT01245959 was done at 10 centers in China, collecting 480 cases treated with CCRT+ICT or CCRT. In our study, we used all the patients treated at our center (Sun Yat-sen University Cancer Center, Guangzhou, China) of this trial as the validation cohort (n = 248).

**Reference**

1. Edge S B, American Joint Committee on Cancer. AJCC cancer staging handbook: from the AJCC cancer staging manual. New York: Springer, 2010.

2. Sun Y, Li WF, Chen NY, Zhang N, Hu GQ, Xie FY, Sun Y, Chen XZ, Li JG, Zhu XD: Induction chemotherapy plus concurrent chemoradiotherapy versus concurrent chemoradiotherapy alone in locoregionally advanced nasopharyngeal carcinoma: a phase 3, multicentre, randomised controlled trial. The Lancet Oncology 2016, 17(11):1509-1520.
