# Supplementary material for: Development and validation of a novel MR imaging predictor of response to induction chemotherapy in locoregionally advanced nasopharyngeal cancer: a randomized controlled trial substudy (NCT01245959)
Source: BMC Med. 2019 Oct 23;17:190. doi: 10.1186/s12916-019-1422-6 (PMC6806559; doi:10.1186/s12916-019-1422-6)
Supplement: Supplementary file 2 — Additional file 2. Magnetic resonance image acquisition parameters. [file 12916_2019_1422_MOESM2_ESM.docx]

**Appendix S2: Magnetic resonance images acquisition parameters**

MR images of each patient were acquired from one of the following Healthcare System platforms ([Discovery MR750, Discovery MR750w, Espree, Signa EXCITE, Signa HDx; GE, USA], [Achieva, Panorama HFO; Philips, Netherlands], or [TrioTim; Siemens, Germany]). The magnetic strengths of the above scanners were 1.0T, 1.5T, and 3.0T, respectively.

During the image acquisition, patients were scanned from the suprasellar cistern to the inferior margin of the sternal end of the clavicle examined, with a head-and-neck combined coil. T1-weighted fast spin-echo images in the axial, coronal, and sagittal planes; and T2-weighted fast spin-echo MR images in the axial plane were obtained before injection of contrast material. At approximately 40 seconds after intravenous Gd-DTPA injection at a dose of 0.1 mmol/kg body weight (Magnevist; Schering, Berlin, Germany), spin-echo T1-weighted axial and sagittal sequences, and spin-echo T1-weighted fat-suppressed coronal sequences were performed sequentially. The acquisition parameters are shown in **Table S1** and **Table S2**.
