# Supplementary material for: Development and validation of a novel MR imaging predictor of response to induction chemotherapy in locoregionally advanced nasopharyngeal cancer: a randomized controlled trial substudy (NCT01245959)
Source: BMC Med. 2019 Oct 23;17:190. doi: 10.1186/s12916-019-1422-6 (PMC6806559; doi:10.1186/s12916-019-1422-6)
Supplement: Supplementary file 3 — Additional file 3. Supplementary methods. [file 12916_2019_1422_MOESM3_ESM.docx]

**Appendix S3: Supplementary methods**

**Inverse probability of treatment weighting (IPTW)**

Age, sex, pEBV-DNA, cervical nodal necrosis, volume, N stage, and T stage were considered in the propensity score model development. First, the above factors were all included in the logistic regression model to predict the choice tendency of ICT+CCT and CCRT. Second, for each continuous variable, the model in the first step was compared with a model that incorporated the restricted cubic spline function with three knots for all continuous variables.

Standardized differences were used to estimate the balance between treatment groups in the unweighted and weighted samples [1]. A standardized difference exceeding 10% means an imbalance in a covariate between two groups [2, 3].

**Image pre-processing**

1. **Interpolation**

Several studies have shown that the in-plane voxel size has an impact on some radiomic features [4]. There exist a number of in-plane pixel pitch in all MR image (0.51 + 0.08 mm). In order to reduce this kind of bias, we adjusted in-plane voxel size of MR image to 0.5 mm using bilinear interpolation.

1. **Image intensity normalization**

The extracted features in texture analysis and quantitative analysis of MRI images may be incomparable between consecutive or repeated scans or, within the same scan but between different anatomic regions. Therefore, normalization should be performed to control inter-scanner and inter-vendor variability of features. We used a histogram matching method for normalization. All image intensities were normalized according to the following equation after the bilinear interpolation [5]:

$$I_{1}=1000\times\frac{I}{I_{0.85}}$$

$$I_{new}=\left\{ \begin{matrix} 0 , I_{1}\leq0 \\ I_{1} , 0<I_{1}<2048 \\ 2048 , I_{1}\geq2048 \end{matrix} \right.$$

where *I* denotes an MR image of a subject and $I_{0.85}$ denotes the value of the first 85% of *I*.

1. **Image filtering**

Wavelet filtering is performed in each image to extract radiomic features different frequency domains. Separable filtering was used to avoid the multi-dimensional convolution. The convolution was performed with a low-/high-pass “Coiflet 1” wavelet filter along the x-/y-direction, separately. L and H denote low-pass and high-pass function, respectively, while X denotes the original image. The wavelet decompositions of X are labeled as XLL, XLH, XHL, and XHH.

**Radiomic features extraction**

The design of radiomic features referred to the image biomarker standardisation initiative (IBSI) [6] and features proposed by Aerts et al. 2014 [7]. The extracted radiomic features can be divided into four groups: first order statistics, shape and size-based features, textural features, and wavelet features. All feature extraction methods were implemented using MatLab 2017b (Mathworks, Natick, MA, USA).

First-order statistics characterized the distribution of intensity values, according to commonly used and basic metrics. Seventeen first-order statistics were adopted, such as energy, entropy, skewness, kurtosis, mean, maximum, and minimum.

Eight descriptors of the two-dimensional size and shape of the tumor region were contained in shape-and-size-based features. This type of feature consists of maximum two-dimensional diameter, circular disproportion, circularity, perimeter, perimeter to area ratio, area compactness1, and compactness2.

The extraction of 3D textural features requires the same voxel size in the three directions (x, y, and z direction). However, the image thickness in z direction (5.11 ± 0.234 mm) was much larger than the in-plane voxel size in x and y direction (0.5mm after normalization). Therefore, texture matrices were determined considering transverse 8-connected voxels. Multi-slice 2D textural features comprise two typical matrices: the gray-level co-occurrence matrix (GLCM) and the gray-level run-length texture matrix (GLRLM). GLCM describes the distribution of co-occurring pixel values of an image region at a given offset while gray GLRLM represents patterns or the spatial distribution of voxel intensities.

We performed “Coiflet 1” wavelet filtering on each MR image and re-extracted the above first order statistics and textural features on each decomposition.

Note that, during the feature extraction, a fixed bin number discretization method with a bin size of 25 was used [6, 7].

**The modified covariate method**

Let T = ±1 be the binary treatment indicator and Y^(1)^ and Y^(−1)^ be the potential outcome if the patient received treatment T = 1 and −1, respectively. P(T = 1) = P(T = −1) = 1/2 without the effects of observed confounding. Fit the following Cox regression model with modified covariates:

$$h\left( t|\boldsymbol{Z},T \right)=h_{0}(t)e^{\gamma\boldsymbol{W}^{*}}$$

where$h(t|\cdot)$ is the hazard function for survival time $\check{X}$while *h*_0_(.) is a baseline hazard function free of covariates ***Z*** and *T*, $\boldsymbol{W}^{*}=\boldsymbol{W}*T/2$. If above formula is correctly specified, then

$$\Delta\left( z \right)=\frac{E\{I_{0}(\check{X}^{(1)})|\boldsymbol{Z=z}\}}{E\{I_{0}(\check{X}^{(-1)})|\boldsymbol{Z=z}\}}=exp\{\gamma\boldsymbol{W(z)}\}$$

$\gamma\boldsymbol{W(z)}$ can be used to stratify the patient population according to $\Delta\left( z \right)$, where $I_{0}\left( t \right)=\int_{0}^{t} h_{0}(u)du$ is a monotone increasing function.

**R packages for data analysis**

“psych” package was used to calculate inter-class correlation coefficients (ICCs) between features and “glm” function was used to get the propensity score of each patient. Survival analysis was conducted by “prodlim,” “prodlim,” and “survcomp” packages.

**Reference**

1. Austin PC and Stuart EA. Moving towards best practice when using inverse probability of treatment weighting (IPTW) using the propensity score to estimate causal treatment effects in observational studies. Stat Med 2015; 34: 3661–3679.

2. Normand ST, Landrum MB, Guadagnoli E, Ayanian JZ, Ryan Tl, Cleary PD, McNeil BJ. Validating recommendations for coronary angiography following acute myocardial infarction in the elderly: a matched analysis using propensity scores. Journal of Clinical Epidemiology 2001; 54(4):387-398.

3. Mamdani M, Sykora K, Li P, Normand SL, Streiner DL, Austin PC, Rochon PA, Anderson GM. Reader’s guide to critical appraisal of cohort studies: 2. Assessing potential for confounding. British Medical Journal 2005; 330(7497):960-962.

4. Limkin, E. J., - Limkin, Elaine Johanna, Reuzé, Sylvain, Carré, Alexandre and et al. "The complexity of tumor shape, spiculatedness, correlates with tumor radiomic shape features." Scientific Reports 2019; 9(1): 4329.

5. Nyul L G, Udupa J K, Zhang X. New variants of a method of MRI scale standardization. IEEE Transactions on Medical Imaging, 2000, 19(2):143-150.

6. Zwanenburg A, Leger S, Vallières M, et al. Image biomarker standardisation initiative. arXiv preprint arXiv:1612.07003, 2016.

7. Aerts HJ, Velazquez ER, Leijenaar RT, Parmar C, Grossmann P, Carvalho S, Bussink J, Monshouwer R, Haibe-Kains B, Rietveld D et al: Decoding tumour phenotype by noninvasive imaging using a quantitative radiomics approach. Nature communications 2014, 5:4006.
