# Supplementary material for: Development and validation of a novel MR imaging predictor of response to induction chemotherapy in locoregionally advanced nasopharyngeal cancer: a randomized controlled trial substudy (NCT01245959)
Source: BMC Med. 2019 Oct 23;17:190. doi: 10.1186/s12916-019-1422-6 (PMC6806559; doi:10.1186/s12916-019-1422-6)
Supplement: Supplementary file 4 — Additional file 4. ICTOS calculation formula. [file 12916_2019_1422_MOESM4_ESM.docx]

**Appendix S4: ICTOS calculation formula**

After feature selection, skewness, variance from GLCM (GLCM_variance), and Long Run High Gray Level Emphasis from GLRLM (GLRLM_LRHGLE) were identified by a multivariable Cox proportional hazards model using the modified covariate method. Skewness is a measure of the direction and degree of skewness of statistical data distribution. GLCM_variance and GLRLM_LRHGLE reflect the tumor’s texture.

The ICTOS model could be obtained using:

$$ICTOS=-0.688*skewness-0.442*GLCM\_variance+0.410*GLRLM\_LRHGLE$$

, where skewness was obtained from the first order statistics; and GLCM_variance and GLRLM_LRHGLE are texture features filtered by X_LH_. The three features were all derived from the contrast enhanced T1-weighted images and were standardized by the z-score method (mean and standard deviation of skewness: -0.829 and 0.449, GLCM_variance: 10.373 and 1.396, GLRLM_LRHGLE: 588.932 and 56.297).

Detailed definitions of the selected features are as follows:

Let:

*X* denote the three dimensional image matrix with *N* voxels,

$P\left( i,j \right)$ be the GLCM for distance $\delta=1$ and direction $\alpha$ (0^o^, 45^o^, 90^o^, 135^o^),

$N_{g}$ be the number of discrete intensity levels in the image,

$\mu$ be the mean of $P\left( i,j \right)$,

$p(i,j|\theta)$ be the $\left( i,j \right)$th entry in the given GLRLM $p$ for a direction $\theta$ (0^o^, 45^o^, 90^o^, 135^o^),

$N_{r}$ be the number of different run lengths.

**Skewness:**

$$skewness=\frac{\frac{1}{N}\sum_{i=1}^{N} {(X\left( i \right)-\bar{X})}^{3}}{{(\sqrt{\frac{1}{N}\sum_{i=1}^{N} {(X\left( i \right)-\bar{X})}^{2}})}^{3}}$$

where $\bar{X}$ is the mean of *X*.

**GLCM_variance:**

$$GLCM\_variance= \sum_{i=1}^{N_{g}} \sum_{j=1}^{N_{g}} \left( i-\mu\right)^{2}P(i,j)$$

**GLRLM_LRHGLE:**

$$GLRLM\_LRHGLE= \frac{\sum_{i=1}^{N_{g}} \sum_{j=1}^{N_{r}} p(i,j|\theta)i^{2}j^{2}}{\sum_{i=1}^{N_{g}} \sum_{j=1}^{N_{r}} p(i,j|\theta)}$$
