# Supplementary figures and images for: Development and validation of a novel MR imaging predictor of response to induction chemotherapy in locoregionally advanced nasopharyngeal cancer: a randomized controlled trial substudy (NCT01245959)
Source: BMC Med. 2019 Oct 23;17:190. doi: 10.1186/s12916-019-1422-6 (PMC6806559; doi:10.1186/s12916-019-1422-6)

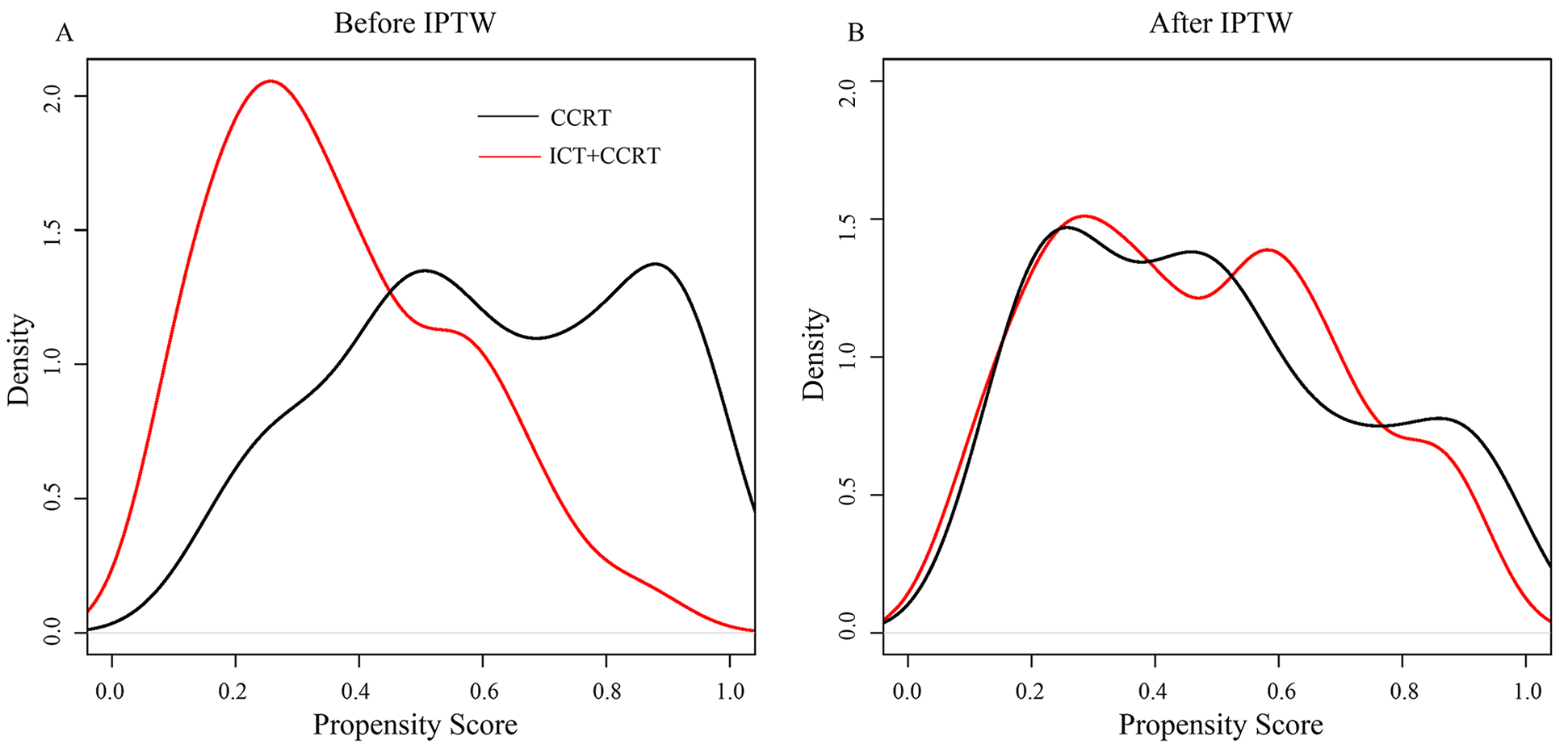

Supplement: Supplementary file 5 — Additional file 5: Figure S1. Performance of inverse probability of treatment weighting between ICT+CCRT and CCRT patient groups. Abbreviations: CCRT, concurrent chemoradiotherapy; ICT, induction chemotherapy; IPTW, Inverse Probability of Treatment Weighting. [file 12916_2019_1422_MOESM5_ESM.tif]

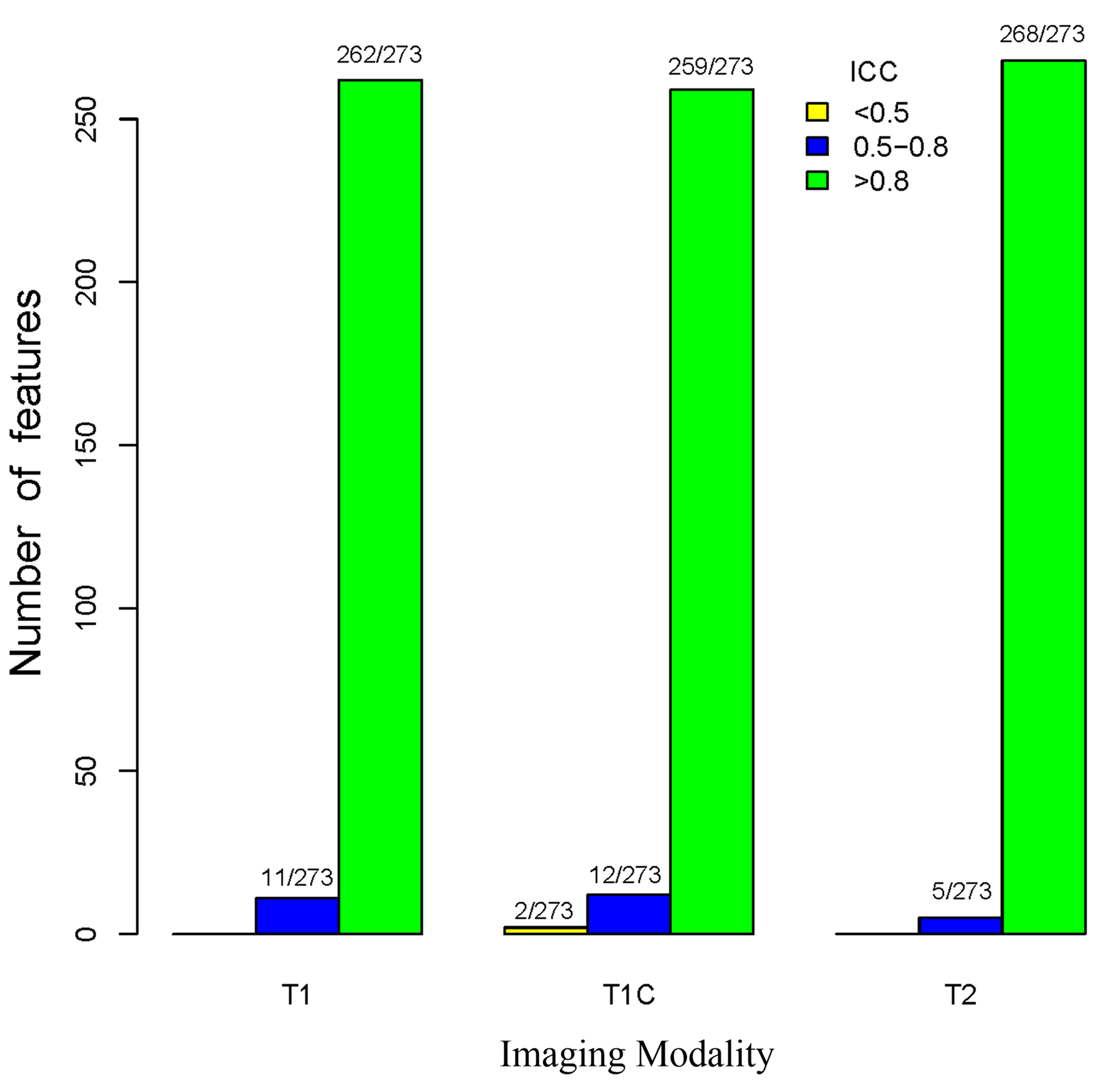

Supplement: Supplementary file 6 — Additional file 6: Figure S2. Analysis of radiomic features’ robustness. Abbreviations: T1, T1-weighted images; T2, T2-weighted images; T1C, contrast enhanced T1-weighted images; ICC, inter-class correlation coefficient. Note: An ICC greater than 0.8 indicates good consistency. As the figure shown, a large majority of features achieve this standard, which affirm the reproducibility of imaging features. [file 12916_2019_1422_MOESM6_ESM.tif]

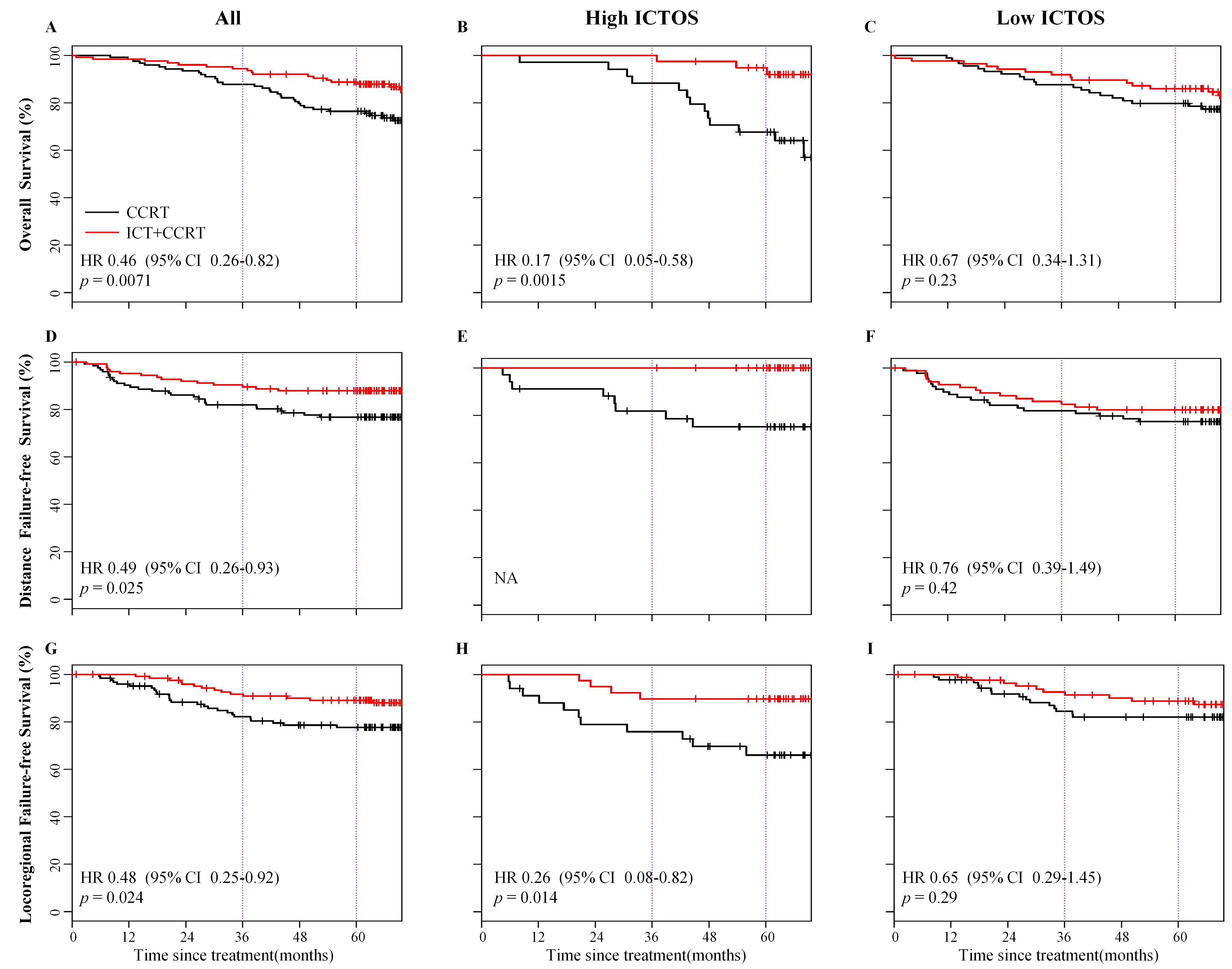

Supplement: Supplementary file 7 — Additional file 7: Figure S3. Kaplan-Meier survival curves with secondary endpoints for the two treatment groups in the validation cohort. Abbreviations: ICT, induction chemotherapy; CCRT, concurrent chemoradiotherapy; ICTOS, Induction Chemotherapy Outcomes Score; HR, hazard ratio; CI, confidence interval. Note: Because there was no patient with distance failure in the high ICTOS group, relative measurements could not be calculated (E). [file 12916_2019_1422_MOESM7_ESM.tif]

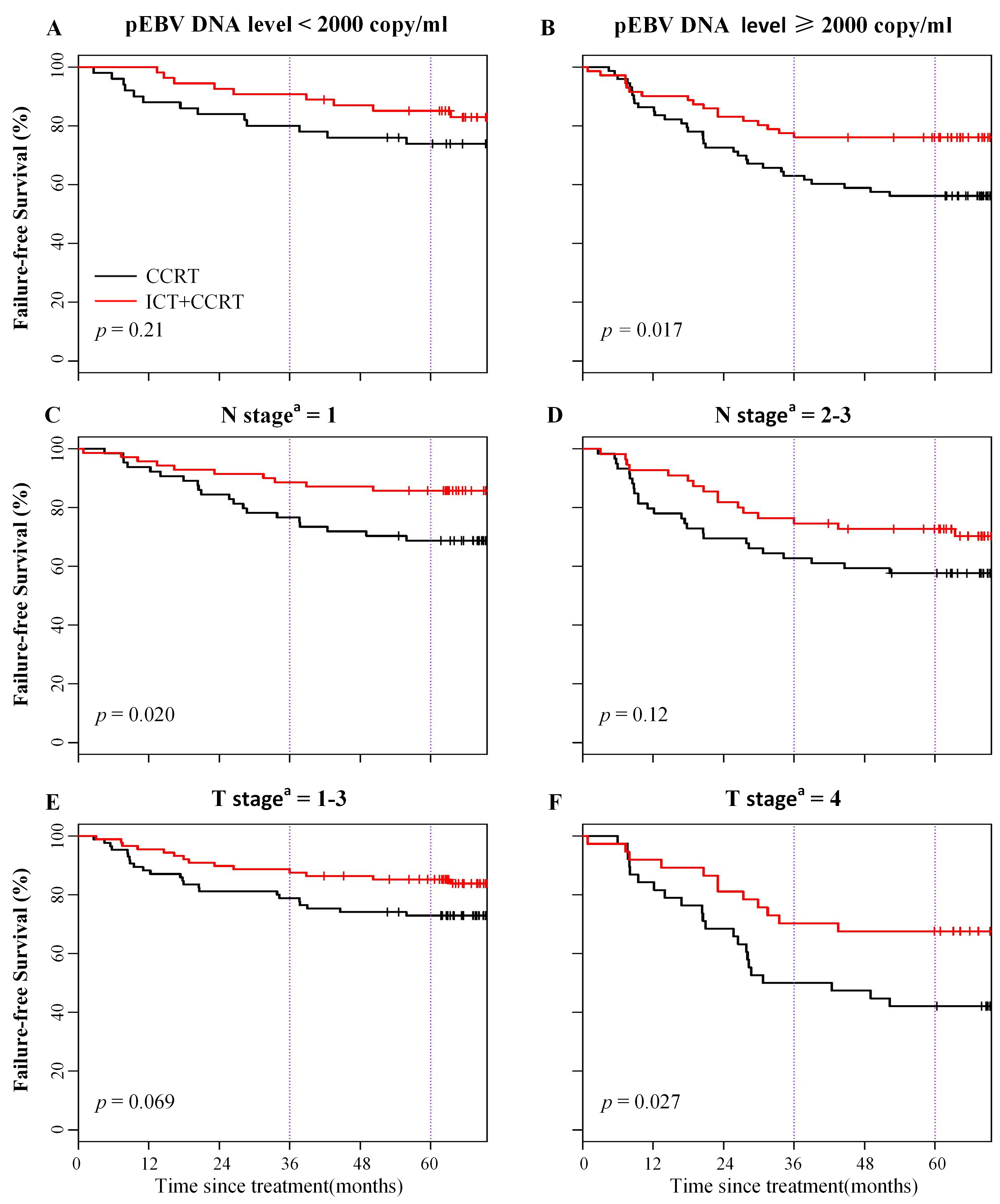

Supplement: Supplementary file 8 — Additional file 8: Figure S4. Failure-free survival in the validation cohort stratified by the usual clinical risk factors. Abbreviations: ICT, induction chemotherapy; CCRT, concurrent chemoradiotherapy; pEBV DNA, plasma Epstein–Barr Virus DNA. Note: Pinteraction = 0.74 for pEBV DNA, Pinteraction = 0.47 for N stage, Pinteraction = 0.77 for T stage. aStaging, T classification, N classification were determined based on the 7th edition of the American Joint Commission on Cancer staging system. [file 12916_2019_1422_MOESM8_ESM.tif]

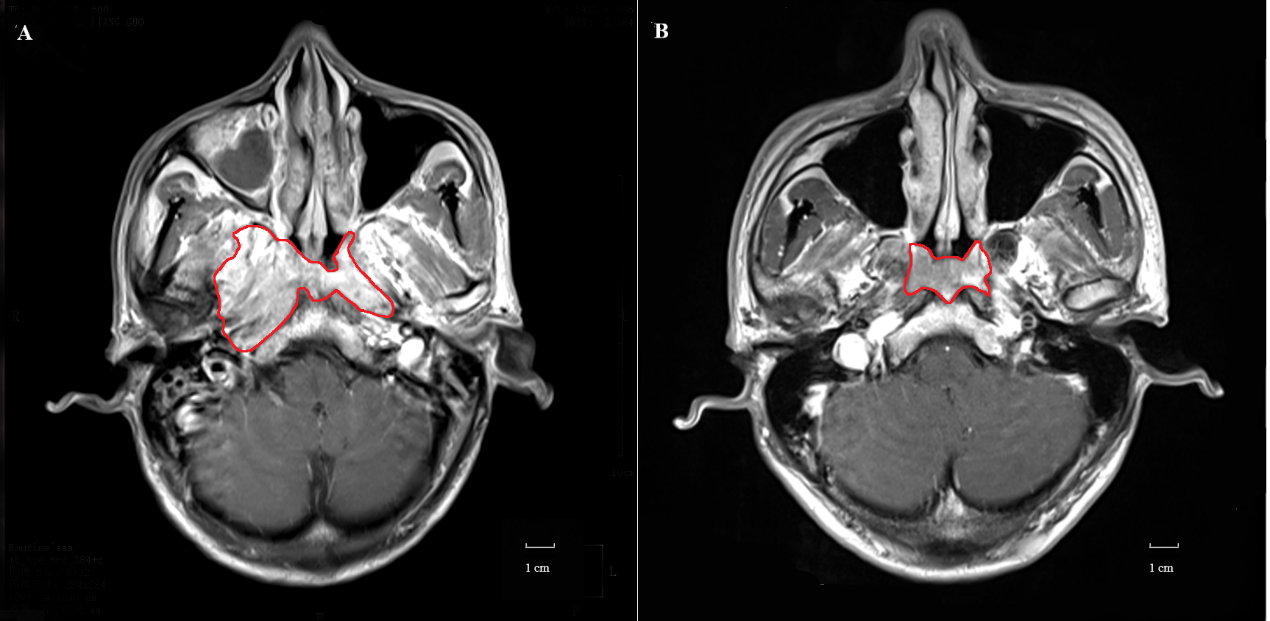

Supplement: Supplementary file 9 — Additional file 9: Figure S5. MR images of patients with high ICTOS and low ICTOS. Abbreviations: MR, Magnetic Resonance; ICTOS, Induction Chemotherapy Outcomes Score. Note: Contrast enhanced T1 MRI images in a patient with high ICTOS (skewness = -2.02; GLRLM_LRHGLE = 4.58; GLCM_variance = -1.26, ICTOS = 3.80, Figure A); and a patient with low ICTOS (skewness = 1.82; GLRLM_LRHGLE = -1.46; GLCM_variance = 1.37, ICTOS = -2.49, Fig. B). [file 12916_2019_1422_MOESM9_ESM.tif]
