# Supplementary material for: Development and validation of a novel MR imaging predictor of response to induction chemotherapy in locoregionally advanced nasopharyngeal cancer: a randomized controlled trial substudy (NCT01245959)
Source: BMC Med. 2019 Oct 23;17:190. doi: 10.1186/s12916-019-1422-6 (PMC6806559; doi:10.1186/s12916-019-1422-6)
Supplement: Supplementary file 10 — Additional file 10: Table S1. MR imaging sequences. [file 12916_2019_1422_MOESM10_ESM.docx]

**Table S1. MR imaging sequences**

| Sequence | TR  (ms) | TE  (ms) | FOV  (mm) | Slice thickness (mm) |
| --- | --- | --- | --- | --- |
| axial T1W FSE | 400–800 | 6-15 | 200×200-260×260 | 5-6.5mm |
| axial T2W FSE | 2000–8000 | 80-120 | 200×200-260×260 | 5-6.5mm |
| axial CE-T1W FSE | 300–1000 | 6-15 | 200×200-260×260 | 5-6.5mm |

*Abbreviations:* TR, repetition time; TE, echo time; FOV, field of view; T1W FSE, T1-weighted fast spin-echo sequence; T2W FSE, T2-weighted fast spin-echo sequence; CE-T1W FSE, T1-weighted contrast-enhanced fast spin-echo sequence.
