# Supplementary material for: Development and validation of a novel MR imaging predictor of response to induction chemotherapy in locoregionally advanced nasopharyngeal cancer: a randomized controlled trial substudy (NCT01245959)
Source: BMC Med. 2019 Oct 23;17:190. doi: 10.1186/s12916-019-1422-6 (PMC6806559; doi:10.1186/s12916-019-1422-6)
Supplement: Supplementary file 11 — Additional file 11: Table S2. Acquisitions parameters of axial CE-T1W FSE in the training and validation cohorts. [file 12916_2019_1422_MOESM11_ESM.docx]

**Table S2. Acquisition parameters of axial CE-T1W FSE in the training and validation cohorts.**

| **Scanner** | **Magnetic field strength (T)** | **Training Cohort**  **(n = 245)** | | | | | | | **Validation Cohort**  **(n = 248)** | | | | | | | |
| --- | --- | --- | --- | --- | --- | --- | --- | --- | --- | --- | --- | --- | --- | --- | --- | --- |
|  |  | **n**  **(%)** | **TR (ms)** | **TE (ms)** | **In-plane voxel size**  **(mm)** |  | **FOV (mm)** | **Image thickness (mm)** | **n**  **(%)** | **TR (ms)** | **TE (ms)** | **In-plane voxel size**  **(mm)** |  | **FOV (mm)** | **Image thickness (mm)** |  |
|  |  |  | **Median (IQR)** | | |  | **Range** | |  | **Median (IQR)** | | |  | **Range** | |  |
| Panorama HFO  (Philips) | 1.0 | 20 (7.9%) | 639.6  (639.6-639.6) | 7.1  (7.1-7.1) | 0.60  (0.58-0.60) |  | 200×200-250×250 | 5.0 | 6  (2.4%) | 583.1  (583.1-625.5) | 7.8  (7.8-10.0) | 0.60  (0.58-0.60) |  | 200×200-240×240 | 5.0 |  |
| Signa HDx\ HDxt  (General Electric) | 1.5 | 23  (9.1%) | 466.7  (333.3-508.3) | 10.0  (8.7-11.0) | 0.43  (0.43-0.45) |  | 200×200-260×260 | 5.0-6.0 | 36  (14.5%) | 483.3  (466.7-583.3) | 9.0  (8.8-11.1) | 0.43  (0.43-0.45) |  | 200×200-240×240 | 5.0-5.5 |  |
| SIGNA EXCITE  (General Electric) | 1.5 | 85  (33.4%) | 520.0  (480.0-520.0) | 10.1  (9.5-10.1) | 0.43  (0.43-0.45) |  | 200×200-260×260 | 5.0 | 104  (41.9%) | 520.0  (480.0-520.0) | 10.1  (10.1-10.2) | 0.43  (0.43-0.45) |  | 200×200-260×260 | 5.0 |  |
| Espree  (General Electric | 1.5 | 12  (4.7%) | 458.5  (457.0-460.0) | 9.8  (9.8-9.8) | 0.60  (0.60-0.60) |  | 230×230 | 5.0 | 5  (2.0%) | 460.0  (460.0-460.0) | 9.8  (9.8-9.8) | 0.60  (0.60-0.60) |  | 230×230 | 5.0 |  |
| TrioTim (Siemens) | 3.0 | 87  (34.3%) | 651.0  (577.0-703.5) | 9.3  (8.8-9.4) | 0.57  (0.57-0.63) |  | 210×210-260×260 | 5.0-6.0 | 97  (39.1%) | 640.0  (600.0-700.0) | 9.3  (8.8-9.4) | 0.57  (0.57-0.63) |  | 210×210-260×260 | 5.0-6.5 |  |
| Achieva  (Philips) | 3.0 | 18  (7.0%) | 610.2  (578.3-610.2) | 8.0  (8.0-8.0) | 0.46  (0.41-0.59) |  | 220×220-240×240 | 5.0 | 0 | - | - |  |  | - |  |  |
| Discovery MR750\750w  (General Electric) | 3.0 | 9  (3.5%) | 645.0  (615.0-677.0) | 8.4  (6.8-8.8) | 0.45  (0.45-0.47) |  | 220×220-240×240 | 5.0 | 0 | - | - |  |  | - |  |  |

*Abbreviations:* CE-T1W FSE, Contrast-enhanced T1-weighted fast spin-echo sequence; TR, repetition time; TE, echo time; FOV, field of view; IQR, interquartile range; n, number; ICTOS, Induction Chemotherapy Outcome Score.

*Note:* Because all the features selected to construct ICTOS were from the axial CE-T1W FSE sequence, the acquisition parameters of this sequence are displayed.
