# Supplementary material for: Development and validation of a novel MR imaging predictor of response to induction chemotherapy in locoregionally advanced nasopharyngeal cancer: a randomized controlled trial substudy (NCT01245959)
Source: BMC Med. 2019 Oct 23;17:190. doi: 10.1186/s12916-019-1422-6 (PMC6806559; doi:10.1186/s12916-019-1422-6)
Supplement: Supplementary file 12 — Additional file 12: Table S3. Standardized differences in the training sets after weighting by propensity score. [file 12916_2019_1422_MOESM12_ESM.docx]

**Table S3. Standardized differences in the training sets after weighting by propensity score**

| Characteristics | Unweighted | Weighted |
| --- | --- | --- |
| Age (< 42 years) | -21.07 | -3.12 |
| Sex (Female) | 18.34 | 7.33 |
| T stage^a^ |  |  |
| T1 | -21.44 | -6.30 |
| T2 | -10.00 | -1.44 |
| T3 | 46.66 | 0.56 |
| T4 | -35.71 | 2.06 |
| N stage^a^ |  |  |
| N1 | 67.54 | 4.01 |
| N2 | -13.45 | 2.67 |
| N3 | -69.60 | -8.94 |
| Lower neck lymph node involvement (yes) | -54.95 | 1.36 |
| Cervical nodal necrosis (yes) | -42.54 | -11.63 |
| Primary tumor volume | -27.90 | -6.65 |
| Pretreatment pEBV DNA level^b^ | -48.54 | -7.60 |

Abbreviations: pEBV DNA: plasma Epstein–Barr Virus DNA;

Note: ^a^Staging, T classification, N classification were determined based on the 7th edition of the American Joint Committee on Cancer/International Union Against Cancer staging system.

^b^Pretreatment pEBV DNA level was added by 1 and inputted into natural logarithm.

For cervical nodal necrosis, although the standardized differences between the groups were greater than 10% after weighting, the absolute differences were small (4.9%).
