# Supplementary material for: Development and validation of a novel MR imaging predictor of response to induction chemotherapy in locoregionally advanced nasopharyngeal cancer: a randomized controlled trial substudy (NCT01245959)
Source: BMC Med. 2019 Oct 23;17:190. doi: 10.1186/s12916-019-1422-6 (PMC6806559; doi:10.1186/s12916-019-1422-6)
Supplement: Supplementary file 13 — Additional file 13: Table S4. Univariate analyses of risk factors in patients with low ICTOS (n = 175). [file 12916_2019_1422_MOESM13_ESM.docx]

**Table S4. Univariate analyses of risk factors in patients with low ICTOS (*n* = 175).**

|  | | **Hazards ratio (95% CI)** | ***P* value** |
| --- | --- | --- | --- |
| **Failure-free survival** | |  |  |
|  | Sex, female *vs.* male | 1.01 (0.98 - 1.05) | 0.40 |
|  | Age, $\geq$ 42 years *vs.* < 42 years | 1.53 (0.87 – 2.70) | 0.14 |
|  | T stage^a^, T4 *vs.* T1-3 | 2.10 (1.20 - 3.68) | 0.0075^*^ |
|  | N stage^a^, N2-3 *vs.* N1 | 1.72 (0.99 – 2.98) | 0.052^*^ |
|  | Pretreatment pEBV DNA level,  $\geq$ 2000 copy/ml *vs.* < 2000 copy/ml | 1.88 (1.05 - 3.37) | 0.030^*^ |
|  | Primary tumor volume, $\geq$ 34ml *vs.* < 34ml | 2.65 (1.46 - 4.78) | < 0.001^*^ |

Abbreviations: 95% CI, 95% confidence interval; pEBV DNA, plasma Epstein–Barr Virus DNA; ICTOS, Induction Chemotherapy Outcome Score; CI, confidence interval.

Note: ^a^Staging, T classification, N classification were determined based on the 7th edition of American Joint Committee on Cancer/International Union Against Cancer staging system.

*P* values were calculated with univariate Cox proportional-hazards model.
